# Inferring Interactivity From Gaze Patterns During Triadic Person-Object-Agent Interactions

**DOI:** 10.3389/fpsyg.2019.01913

**Published:** 2019-08-21

**Authors:** Mathis Jording, Arne Hartz, Gary Bente, Martin Schulte-Rüther, Kai Vogeley

**Affiliations:** ^1^Cognitive Neuroscience, Institute of Neuroscience and Medicine (INM-3), Research Center Jülich, Jülich, Germany; ^2^Department of Psychiatry, University Hospital Cologne, Cologne, Germany; ^3^JARA-BRAIN, Aachen, Germany; ^4^Translational Brain Research in Psychiatry and Neurology, Department of Child and Adolescent Psychiatry, Psychosomatics, and Psychotherapy, RWTH Aachen University, Aachen, Germany; ^5^Department of Communication, Michigan State University, East Lansing, MI, United States

**Keywords:** social gaze, joint attention, eye contact, triadic interaction, non-verbal communication, social psychology, human-agent interaction

## Abstract

Observing others’ gaze informs us about relevant matters in the environment. Humans’ sensitivity to gaze cues and our ability to use this information to focus our own attention is crucial to learning, social coordination, and survival. Gaze can also be a deliberate social signal which captures and directs the gaze of others toward an object of interest. In the current study, we investigated whether the intention to actively communicate one’s own attentional focus can be inferred from the dynamics of gaze alone. We used a triadic gaze interaction paradigm based on the recently proposed classification of attentional states and respective gaze patterns in person-object-person interactions, the so-called “social gaze space (SGS).” Twenty-eight participants interacted with a computer controlled virtual agent while they assumed to interact with a real human. During the experiment, the virtual agent engaged in various gaze patterns which were determined by the agent’s attentional communicative state, as described by the concept of SGS. After each interaction, participants were asked to judge whether the other person was trying to deliberately interact with them. Results show that participants were able to infer the communicative intention solely from the agent’s gaze behavior. The results substantiate claims about the pivotal role of gaze in social coordination and relationship formation. Our results further reveal that social expectations are reflected in differential responses to the displayed gaze patterns and may be crucial for impression formation during gaze-based interaction. To the best of our knowledge, this is the first study to document the experience of interactivity in continuous and contingent triadic gaze interactions.

## Introduction

During social interactions, we consistently focus on the eyes of our interaction partner because it is the fastest and easiest way to access the inner experience of another person (Yarbus, 1967; Baron-Cohen et al., 1997; Emery, 2000). From the eye region alone we are able to infer age, gender, and personality and even identify individual persons (George and Conty, 2008; Itier and Batty, 2009). We also use gaze to ensure successful communication and smooth interactions by coordinating turn-taking (Argyle and Cook, 1976) and coordinating attention with others. This ability may constitute the phylogenetic and ontogenetic basis of cooperation (Tomasello et al., 2007; Grossmann, 2017). The most prevalent example of coordinated gaze is joint attention i.e., the joint focus of two persons gaze on an object, including gaze following and leading the gaze of others (Emery, 2000). The ability to follow someone else’s gaze toward objects is acquired very early in life, possible starting at the age of 6 months (Senju and Csibra, 2008), it provides the basis for reinforcement learning (Vernetti et al., 2017), and the development of a theory of mind and language (Morales et al., 1998). It is therefore not surprising that the proficiency in gaze following predicts social competence, self-regulation abilities, and even the depth of information processing and IQ (Mundy and Newell, 2007).

During everyday encounters with other people, we do not know in advance whether the person we meet is trying to engage us in an interaction or is merely exploring the environment. In other words, we have to disambiguate the dual function of social gaze (Gobel et al., 2015; Jarick and Kingstone, 2015), or the simultaneous use of gaze for visual perception and for communicating with others. That is, we take the communicative states of others into account and adjust our gaze behavior for social adequacy accordingly (Risko and Kingstone, 2011; Wu et al., 2013). Conversely, this also implies that by observation alone we cannot be sure of whether gaze behavior of others is a communicative signal toward us or merely serves perceptual means. One powerful communicative signal is mutual eye contact (Senju and Johnson, 2009) which increases emotional empathy and modulates attention (Farroni et al., 2002; Senju and Hasegawa, 2005; Dalmaso et al., 2017). Thus, eye contact likely fosters the experience of a connection with another person. Furthermore, attempts to establish joint attention can be considered as prototypical gaze-based interaction. However, as of yet it is unclear, which cues are most informative in disambiguating the dual function of social gaze and inferring social communicative intent based on observed gaze alone.

Here we investigate the human ability to recognize communicative attempts from gaze. Using gaze-contingent paradigms with virtual characters (VC) it is possible to investigate ongoing interactions while retaining full experimental control (Vogeley and Bente, 2010; Wilms et al., 2010; Pfeiffer et al., 2013b; Georgescu et al., 2014; Oberwelland et al., 2016, 2017). However, these paradigms suffer from two major limitations: (1) gaze communication is implemented as a series of short, discrete and isolated events and not as an ongoing flux of interaction; (2) the respective paradigms mostly relied on explicit instructions or repetitive, monotonic, and predictable agent behavior. Resolving these limitations required both a theoretical foundation and technological advancements. Theoretically, we developed a new holistic taxonomy of social gaze, the “social gaze space (SGS)” (Jording et al., 2018). The SGS covers all possible categorical states of attention and interaction during gaze-based triadic interactions (constituted by two interactants and at least one object in a shared environment). The different gaze states include: “partner-oriented (PO),” during which the attention is directed solely on the interaction partner; “object-oriented (OO),” attention directed solely on the object(s) in the environment; “introspective (INT),” attention disengaged from the outside world and directed toward inner (e.g., bodily) experiences; “responding joint attention (RJA),” a state of actively following the partner’s gaze toward objects of his choice; and “initiating joint attention (IJA),” a state in which the partner’s gaze is led toward the objects of one’s own choice. The two joint attention states (RJA and IJA) are interactive states in which the agents’ behavior depends on the interaction partner, whereas the other three describe states of passive observation. Note, that these five states individually describe the behavior of one of the interaction partners. The interaction between both can be characterized as the combination of both individual states toward a “dual state” (Jording et al., 2018).

Technically, we implemented all five different gaze states of the SGS in the gaze-contingent agent-platform “TriPy” (Hartz et al., submitted). Unlike previous agent-systems, it can generate all SGS states including their responsive properties in real-time. The agent allows for mutual interactions in a continuous and immersive, hence, ecologically valid fashion. The agent’s behavior is governed by sets of probabilistic parameters and timing parameters, based on empirical observations during continuous gaze-based interactions (Hartz et al., submitted).

We used this setup to address the question whether and how humans identify communicative intentions from gaze alone. To this end, we asked participants to interact with an algorithmically controlled VC while believing that a real human controlled the VC. Participants had to rate, whether their interaction partner was trying to interact with them or not. We analyzed the participants’ decisions and response times (RT) as well as their gaze behavior and the occurrence of eye contact and instances of joint attention. We were interested whether participants would experience differences in the degree of interactivity of the different gaze states as implied by the SGS. We assumed that from the non-interactive states, PO would be rated the most interactive because here the agent focused on the perceiver proportionately more, increasing the probability of eye contact. With respect to interactive states, we hypothesized that the IJA state might be experienced less frequently as interactive compared to RJA. While in IJA participants need to actively follow the agent in order to learn, whether this would move the agent to “show” them the next object, in RJA the agent would strictly follow the participant which we assumed to be easily noticeable. After the experiment, we let participants rate the difficulty of the task and compared it to their performance in identifying interactive situations as an indicator of the conscious accessibility of the underlying cognitive processes.

## Materials and Methods

### Participants

A total of 28 participants without any record of psychiatric or neurological illnesses were recruited via mailing lists, gave their written consent and were compensated for their participation (10€ per hour). Three participants were excluded due to technical failure (*n* = 1) and lack of conviction to interact with a real person (*n* = 2). Data from 25 participants (aged 19 – 57; mean = 31.08, *SD* = 11.21; 16 identifying as female, 9 as male) were further analyzed. This study was approved by the ethics committee of the Medical Faculty of the University of Cologne, Germany, and strictly adhered to the Declaration of Helsinki and the Principles of Good Scientific Practice.

### Procedure and Tasks

**FIGURE 1:**
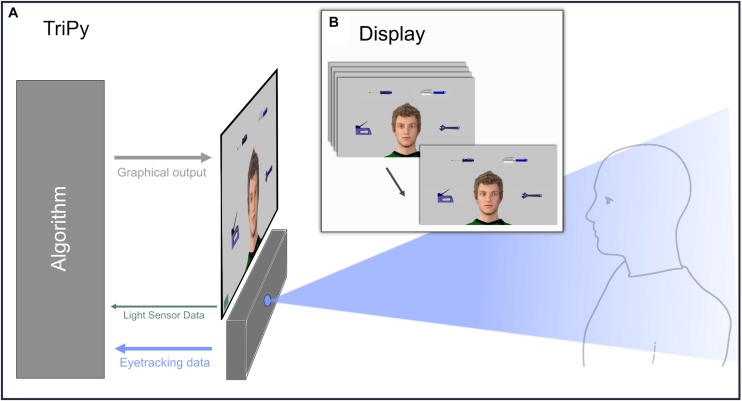
Illustration of the technical setup and the participants’ perspective during the experiment. **(A)** Illustration of a participant interacting with the agent controlled by the platform TriPy. **(B)** The behavior of the agent created by TriPy as seen from the perspective of the participant **(B)**.

Before the experiment, participants were briefly introduced to a confederate of the same sex but were brought to another room where they received the detailed written experimental instructions that were repeated orally. Participants were told that both communication partners would be represented by the same standard male VC serving as avatar and that both could only communicate via gaze behavior. They were further told that they would be seated in front of a monitor that displayed the avatar of their partner representing the partner’s eye movements on the basis of data provided by two identical eye-tracking systems and updating the respective gaze direction of the avatars in real-time (Figure 1A). In fact, participants always solely communicated with an agent controlled by a computer algorithm (Hartz et al., submitted). Participants would further see four trial-wise changing objects, at fixed positions and obviously visible for the partner’s avatar (Figure 1B). Neither the VC nor the objects were shown to the participant before the start of the experiment.

Participants were further instructed to take two different roles: (1) The Observation-Role (ObR), and (2) the Action-Role (AcR). For the ObR condition, there were no trial specific instructions apart from the task to ascertain whether their partner was trying to “interact” or not (German “austauschen” or “interagieren”), “interacting” was defined as an encounter in which both partners respond to the gaze behavior of the partner in a mutual and reciprocal fashion. Participants were asked to answer only as soon as they felt “quite sure” but were reminded that each trial ended at the latest after 30 s and they therefore would have to hurry. The time between beginning of the trial and button press was logged as RT. When participants had not pressed a button within 30 s, they were asked to decide more quickly in the next trial. After each trial, the participant’s choice was displayed on the screen until participants indicated their readiness to continue via button press. Afterward, a message was displayed, asking the participants to wait until their partner was ready for the next trial. This delay was introduced in order to support the participants believe in the confederate based coverstory. The next trial would then begin after a random (uniformly distributed) duration of 1 – 5 s with the appearing of the agents face on the screen.

During the AcR condition, participants were explicitly instructed to engage in one of the states of the SGS (Jording et al., 2018) with the following instructions: “Please concentrate on your partner” (German: “Bitte konzentrieren Sie sich auf Ihren Partner”; PO); “Please attend to the objects” (German: “Bitte achten Sie auf die Objekte”; OO); “Please keep your eyes open and concentrate on your breath” (German: “Bitte lassen Sie Ihre Augen geöffnet und konzentrieren Sie sich auf Ihren Atem”; INT); “Please interact with your partner and let his gaze guide you” (German: “Bitte versuchen Sie sich mit Ihrem Partner auszutauschen und lassen Sie sich von seinem Blick leiten”; RJA), or “Please interact with your partner and use your gaze to guide him” (German: “Bitte versuchen Sie sich mit Ihrem Partner auszutauschen und nutzen Sie Ihren Blick um ihn zu leiten”; IJA). No further instructions were given and participants were told that there was no correct or wrong behavior and they should behave according to their intuitive understanding of these instructions. Trials stopped after 30 s and were followed by a short break of 2 – 6 s.

Whereas ObR was the target condition allowing measuring the experience of interactivity, the AcR condition was included to support the cover story, as participants believed to be interacting with some other real participants and thus would expect a balanced study design with the same tasks for both participants. Both roles were presented alternatingly in three blocks each, with 16 trials per block during ObR and 10 trials per block for AcR. The order of blocks and state instructions within blocks was randomized across participants. After two blocks participants were given a short break of up to 3 min to prevent fatigue and to allow for recalibration of the eyetracker to avoid drifting artifacts.

### Setup, Agent-Platform, and Pilot Study

The setup consisted of an eye-tracker with a sampling rate of 120 Hz and an accuracy of 0.5° (Tobii TX300; Tobii Technology, Stockholm, Sweden). A 23” monitor with a screen resolution of 1920^∗^1080 pixels mounted on top of the eye-tracker was used as display (Figure 1A). Participants were seated at a distance between 50 – 70 cm to the monitor. A PC-keyboard with the marked buttons “J” and “N” was used for participant responses during ObR. A light sensor based system (StimTracker, Cedrus Corporation, San Pedro, CA, United States) ensured that timing of presented stimuli by the algorithm and actual graphical output were in sync.

**FIGURE 2:**
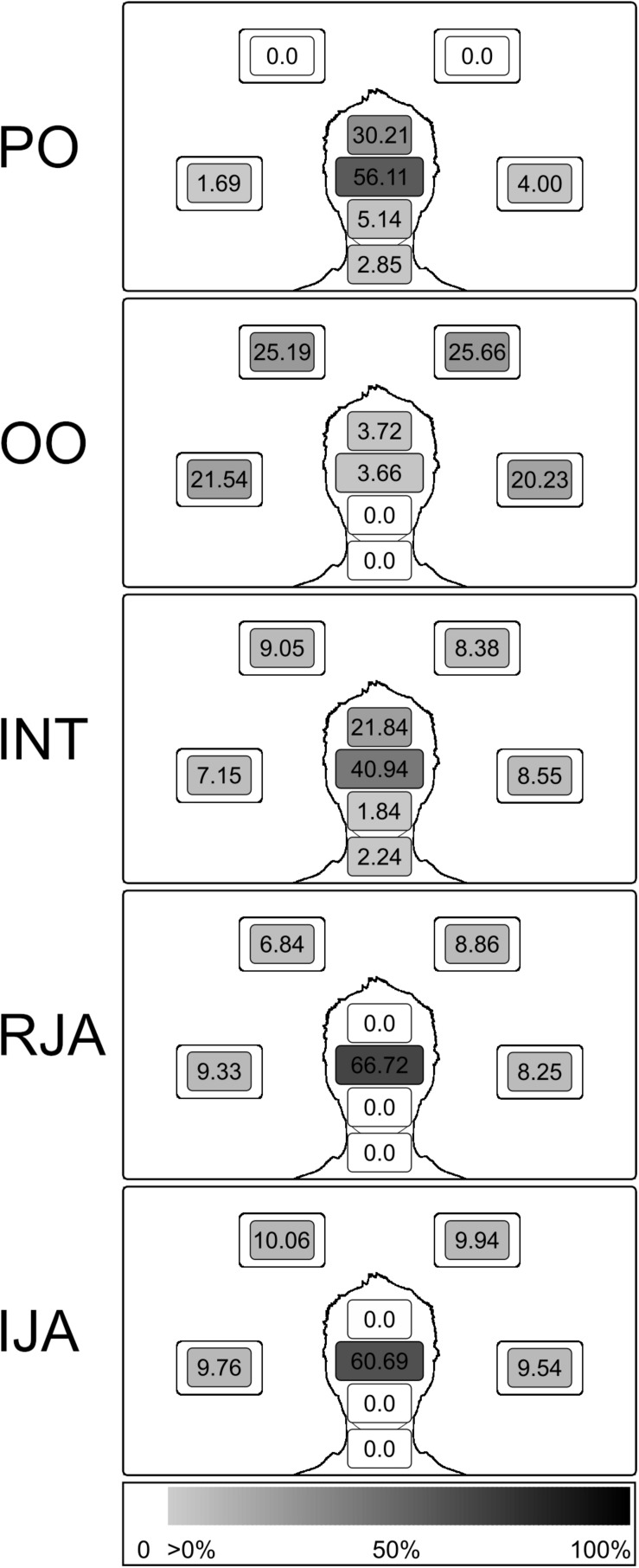
Illustration of the distribution of the agent’s visual attention separately cumulated for the different gaze states. Numbers express the rate in percent with which the agent looked at the AoIs in total in the specific state as portion of all fixations, color schemes coding serve as additional illustration (white, AoIs not being targeted; light gray, low rate; black, high rate; see color bar legend at the bottom).

The agent’s behavior and graphical output was controlled by the agent-platform “TriPy” (Hartz et al., submitted), implemented in Python 2.7 (Python Software Foundation^1^) using PyGaze (Dalmaijer et al., 2014) and PsychoPy (Peirce, 2008). TriPy is based on a gaze-contingent algorithm that adapts the behavior of a VC to the behavior of the participant in real-time (Wilms et al., 2010). In contrast to previous setups, TriPy does not require a prior determination of the exact course and timing of the agents’ behavior. Instead, behavior in the non-interactive states is implemented on a probabilistic basis in which the agent displays different micro states (e.g., a moment of looking at one of the objects) with different probabilities (Figure 2). In the RJA state the agent follows the participants gaze toward the objects and looks back at the eyes of the participant, when being looked at himself, with a randomly drawn offset between 311.06 – 589.93 ms (lognorm distributed, range 6.06 ± 0.32). In the IJA state the agent looks at the participant and as soon as eye contact is established or after a randomly drawn waiting period of 772.78 – 2321.57 ms (lognorm distributed, range 7.2 ± 0.55) looks at one of the objects at random. As soon as the participant follows or after a randomly drawn waiting period of 780.55 – 2440.60 ms (lognorm distributed, range 7.23 ± 0.57), the agent starts anew with trying to establish eye contact and subsequently choosing a new object at random (video examples of the agents behavior in all states can be found in the Supplementary Material). These microstates, their durations and transition probabilities, as well as temporal parameters of the interactive agents’ states were empirically informed by a pilot study (Hartz et al., submitted). The anthropomorphic VC was created with the modeling software Daz Studio 3.1 (DAZ Productions, Inc., United States).

During the ObR condition, the agent equally often displayed either any of the interactive (25% for each of the interactive states RJA and IJA) or any of the non-interactive states (16.67% for each of the non-interactive states PO, OO, and INT). This partitioning ensured that participants encountered interactive and non-interactive states equally often and thus could not exceed a 50% correctness rate by guessing. During AcR – which was established only to let participants continuously believe that they were interacting with the interaction partner to whom they had been introduced before the experiment – the agents’ states corresponded to the states of the participant the agent displayed non-interactive states (PO, OO, or INT) when the participant herself was in a non-interactive state with all combinations of agent and participant states appearing equally often. Each interactive-state of the participant was answered by the agent with the complementary interactive-state (RJA with IJA; IJA with RJA).

### Questionnaires and Post-experimental Inquiry and Information

After the experiment participants filled out a post-experimental questionnaire asking on visual analog scales (ranging from 1 to 6): (A1) how difficult they had experienced the ObR tasks, (A2) how difficult the AcR tasks, (A3) how natural they had experienced the interaction, and (A4) how they rated the quality of the technical realization of the VC’s eye movements. In addition, participants were given the chance to respond in open texts relating to: (B1) their assumptions as to the purpose of the study, (B2) anything that bothered them during tasks of both types ObR and AcR, (B3) any strategies they had employed in their attempt to communicate with the other person, (B4) how the naturalness of the interaction could be improved, (B5) whether there was anything else to the experiment which bothered them. The participants’ belief in the cover story was further tested in an interview by the experimenter. Participants were asked how well the communication with the partner had worked, whether they had considered what their partner was thinking and whether they had tried to empathize with their partner and whether they had applied specific strategies in their communication with the partner. In addition to the post-experimental questionnaire, participants, either before or after the experiment, also answered a demographic questionnaire and the German version of the autism-spectrum-quotient (AQ; Baron-Cohen et al., 2001). However, for none of the participants AQ results pointed toward autistic symptomatology (cut-off > 32; Baron-Cohen et al., 2001). After the experiment, interview, and questionnaires participants were informed about the nature of the cover story and explained its necessity. Now, participants were asked directly, whether they have had any suspicions as to the nature of the experiment or their partner.

### Data Preprocessing and Statistical Analysis

From a total 1200 trials in the ObR condition (25 participants with 48 trials each), 39 trials were excluded due to missing responses or RT exceeding 30 s, another 201 trials were excluded because more than 20% of gaze data were missing due to technical problems, 960 trials remained for analysis. Response, eye-tracking, and questionnaire data were preprocessed and statistically analyzed with R (R Development Core Team, 2008) and RStudio (RStudio Team, 2016). Response and eye-tracking data were analyzed with (generalized) linear mixed effects models, as recommended for data from repeated measures designs (Pinheiro and Bates, 2009), using the lmer() and glmer() function from the lme4 package (Bates et al., 2015). The general influence of predictors was assessed in likelihood ratio tests, comparing how well models including different predictors fit a given data set while taking into account (i.e., penalizing) the models’ complexity. The significance of the effect of each predictor was tested by comparing a model comprising the predictor with the same model without the predictor against a significance level of 0.05. Where likelihood ratio tests revealed significant effects of factors, we conducted Tukey *post hoc* tests for the comparison between all individual factor levels (correcting for multiple comparisons) with the glht() function from the multcomp package (Hothorn et al., 2008).

For the analysis of gaze data we computed “relative fixation durations” as the portion of cumulative fixation durations spent on the AoIs “eyes”, “face” (not including the eyes), or “objects” (the four objects taken together). Instances of eye contact and joint attention were defined as situations in which the participant and the agent both looked at the eyes of the partner (eye contact) or simultaneously at the same object (joint attention). Two consecutive eye contact or joint attention events on the same object were treated as a single continuous event when they were less than 100 ms apart in order to prevent artificial inflation of events due to eye blinks. Only eye contact and joint attention events with a minimum duration of 50 ms were included in the analysis.

Data from the visual analog scales in the post-experimental questionnaire were summarized as group means. In addition, Spearman correlations between participants’ post-experimental self-reports and their task performance were computed. The effect of the participants’ age and gender on their responses were analyzed in linear models. Open text responses and statements from the interview were checked for any indications of mistrust in the cover story (e.g., statements indicating lack of conviction to interact with a real person).

## Results

### Interactivity Ratings

In order to test whether participants were able to correctly identify interactive situations we first compared within ObR the ratings between the non-interactive states (PO, OO, and INT) and the interactive states (RJA and IJA) as a logistic regression with random intercepts for participants. The analysis revealed a highly significant effect on the model fit [*χ*^2^(1) = 222.59, *p* < 0.001]. The chance of being rated as interactive was 27.07% for the non-interactive states and 73.32% for the interactive states, corresponding to a difference in the predicted odds ratio by the factor of 8.45 (*M* = 2.13, *SD* = 0.16).

**FIGURE 3:**
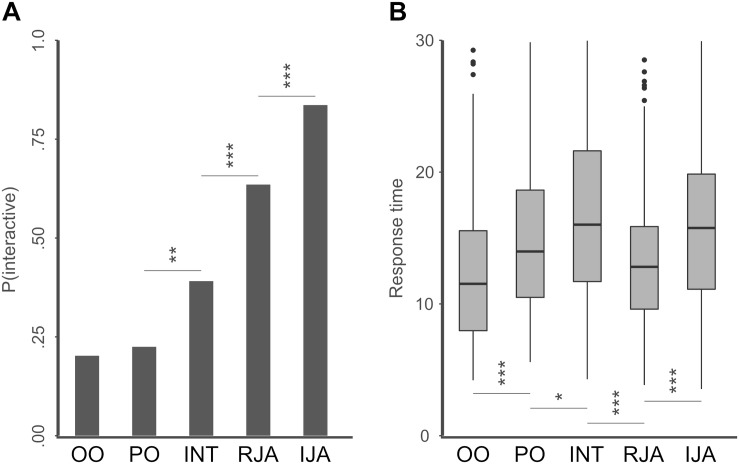
Plots of mean interactivity ratings and mean response times separately for the different gaze states. **(A)** Mean interactivity ratings for different agent states. Asterisks indicate significant differences between neighboring states (when ranked in ascending order) in *post hoc* tests (^∗^ < 0.05; ^∗∗^ < 0.01; and ^*⁣**^ < 0.001). **(B)** Mean RTs in ms for different agent states. Asterisks indicate significant differences between neighboring states (when ranked in ascending order of mean interactivity ratings) in *post hoc* tests (^∗^ < 0.05; ^∗∗^ < 0.01; and ^*⁣**^ < 0.001).

In a next step we looked at the difference between the individual states (Figure 3A), again analyzed as logistic regression with random intercepts for participants. A model comprising the agent state as fixed effects fitted the data significantly better than the null model including only the intercept [*χ*^2^(4) = 266.70, *p* < 0.001]. *Post hoc* tests revealed significantly lower ratings for PO vs. INT (*M* = −0.86, *SD* = 0.26, *z* = −3.30, *p* = 0.009), INT vs. RJA (*M* = −1.06, *SD* = 0.22, *z* = −4.79, *p* < 0.001), and RJA vs. IJA (*M* = −1.13, *SD* = 0.23, *z* = −4.92, *p* < 0.001), but not between OO and PO (*M* = −0.17, *SD* = 0.28, *z* = −0.60, *p* = 0.975). Note that for the sake of simplicity we only report comparisons between neighboring ranks when sorted by mean estimates. All other comparisons between states yielded highly significant differences (all *p* < 0.001).

RTs (Figure 3B), were logarithmized and again analyzed in a linear mixed effects model with random intercepts for subjects. A group-wise comparison between the interactive and the non-interactive states as fixed effects had no significant effect on the model fit [*χ*^2^(1) = 0.36, *p* < 0.55]. However, including the individual agent states in the model as fixed effects proofed to fit the data significantly better than the null model [*χ*^2^(4) = 82.55, *p* < 0.001]. Corresponding to the results from the interactivity ratings, *post hoc* tests revealed significant differences between OO & PO (*M* = −0.18, *SD* = 0.04, *z* = −4.49, *p* < 0.001), PO & INT (*M* = −0.12, *SD* = 0.04, *z* = −2.85, *p* = 0.035), INT & RJA (*M* = 0.22, *SD* = 0.04, *z* = 5.83, *p* < 0.001), and RJA & IJA (*M* = −1.84, *SD* = 0.03, *z* = −5.55, *p* < 0.001). Note that the differences between OO & INT (*M* = −0.30, *SD* = 0.04, *z* = −7.33, *p* < 0.001), PO & RJA (*M* = 0.10, *SD* = 0.04, *z* = 2.748, *p* = 0.048), and OO and IJA (*M* = −0.26, *SD* = 0.04, *z* = −7.17, *p* < 0.001) also reached significance. In order to investigate whether the quality of the participants’ ratings would increase with longer decision time we computed mean correctness scores (RC; correct = “non-interactive” for PO, OO, and INT or “interactive” for RJA and IJA) for each participant. We found a significant relationship between the participants’ mean RC and mean RT (r = 0.45, *p* < 0.05). In addition, we analyzed, whether the participants’ age or gender had an influence on their decisions. However, neither age nor gender had any significant effect on the mean RCs [age: *χ*^2^(1) = 2.21, *p* < 0.151; gender: *χ*^2^(1) = 2.12, *p* < 0.159] or mean RTs [age: *χ*^2^(1) = 0.518, *p* < 0.479; gender: *χ*^2^(1) = 1.43, *p* < 0.245].

### Gaze Behavior

**FIGURE 4:**
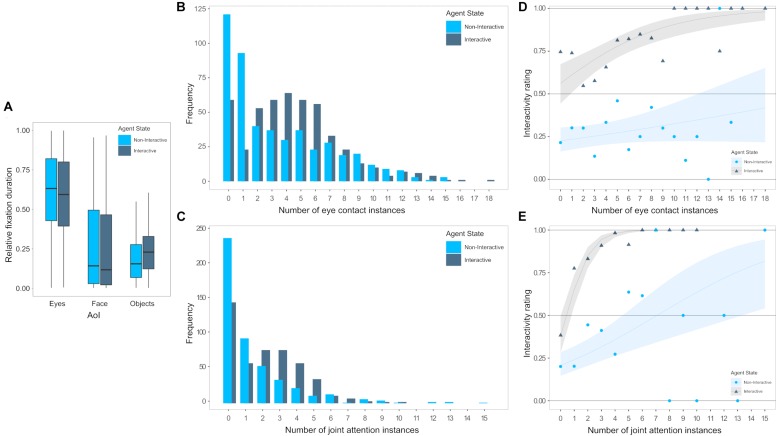
Illustration of the participants gaze behavior and instances of eye contact and joint attention between participant and agent in connection to the participant’s rating of the agents interactivity, separately for an agent behaving non-interactively (light blue) vs. interactively (dark blue). **(A)** Boxplots of relative fixation durations as the portion of time spent on the AoIs Eyes, face, and objects per trial. **(B)** Frequencies of eye contact instances per trial. **(C)** Mean rates (circles and triangles) and model predictions with 95% confidence intervals (lines and ribbons) of interactivity ratings for differing numbers of eye contact instances per trial. **(D)** Frequencies of joint attention instances per trial. **(E)** Mean rates (circles and triangles) and model predictions with 95% confidence intervals (lines and ribbons) of interactivity ratings for differing numbers of joint attention instances per trial.

For the participants’ gaze behavior during ObR, we analyzed the effect of non-interactive vs. interactive states, of the AoIs Eyes, Face and Object and the interaction between states and AoIs on relative durations (proportion of cumulative fixation durations from 0 to 1, Figure 4A). Tests did not reveal significant improvements in model fit for including states [*χ*^2^(1) = 0.00, *p* = 0.994] but for AoI [*χ*^2^(2) = 948.37, *p* < 0.001], and the interaction of state^∗^AoI [*χ*^2^(2) = 12.40, *p* = 0.002]. A *post hoc* test between factor combinations was conducted in order to identify effects potentially driving the interaction. However, corrected for multiple testing, the comparisons between non-interactive and interactive states did not reveal any significant differences for the AoIs Eyes (*M* = −0.03, *SD* = 0.02, *z* = −1.80, *p* = 0.467), Face (*M* = −0.03, *SD* = 0.02, *z* = −1.64, *p* = 0.565), or Objects (*M* = −0.04, *SD* = 0.07, *z* = −2.58, *p* = 0.102).

The effect of a non-interactive vs. interactive agent on the number of instances of eye contact (Figure 4B) and joint attention (Figure 4C) per trial was analyzed in generalized mixed effects models for Poisson distributed data. Including the interactivity of the agent significantly increased model fits for the prediction of the amount of eye contact [*χ*^2^(1) = 68.19, *p* < 0.001] as well as the amount of joint attention instances [*χ*^2^(1) = 72.75, *p* < 0.001]. When the agent behaved interactively, the occurrence of eye contact instances increased by a factor of 1.31 (*M* = 0.27, *SD* = 0.03) and the occurrence of joint attention instances increased by a factor of 1.52 (*M* = 0.42, *SD* = 0.05).

We then analyzed whether the occurrence of instances of eye contact (Figure 4D) or joint attention (Figure 4E) had a predictive value for the participants’ subsequent interactivity rating and whether the prediction would differ depending on the agent behaving either non-interactively or interactively. To this end, we compared linear mixed effects models including the agents’ interactivity, the number of instances of eye contact or joint attention, respectively, as well as the interaction between both. All three, the inclusion of the agents’ interactivity [*χ*^2^(1) = 222.57, *p* < 0.001], the inclusion of the number of eye contact instances [*χ*^2^(1) = 14.86, *p* < 0.001], as well as the interaction between both [*χ*^2^(1) = 9.52, *p* = 0.002], and significantly improved model fits. The predicted probability of the agents′ behavior being rated as interactive increased with the number of eye contact instances (*M* = 0.05, *SD* = 0.03), but this effect was especially strong when the agent actually behaved interactively (*M* = 0.15, *SD* = 0.05). For the analysis of the effect of joint attention, again, the inclusion of the agents’ interactivity [*χ*^2^(1) = 222.59, *p* < 0.001], the inclusion of the number of joint attention instances [*χ*^2^(1) = 96.54, *p* < 0.001], as well as the interaction between both [*χ*^2^(1) = 73.16, *p* < 0.001], significantly improved model fits. Accordingly, the predicted probability of the agents′ behavior being rated interactive increased with the number of joint attention instances (*M* = 0.19, *SD* = 0.05) with an even stronger effect when the agent actually behaved interactively (*M* = 0.92, *SD* = 0.12).

### Questionnaires and Post-experimental Inquiry

In the post experimental inquiry participants reported on the perceived difficulty of the ObR task (*M* = 2.80, *SD* = 1.38) and the AcR task (*M* = 1.76, *SD* = 0.72), the quality of the technical implementation of the agents′ eye movements (*M* = 3.21, *SD* = 0.88), and the naturalness of the interaction (*M* = 2.96, *SD* = 1.30). We compared ratings of the task difficulty to the participants’ mean tendency to experience the agent as interactive, their mean performance (response correctness) as well as mean RTs. Difficulty ratings neither correlated significantly with the participants’ tendency to rate the agent’s behavior as interactive (*r*s = −0.07, *p* > 0.05) nor with their response correctness (*r*s = 0.02, *p* > 0.05) nor with RTs (*r*s = −0.24, *p* > 0.05).

In order to assess effects of autistic traits we compared models comprising and not comprising the AQ scores as predictor. Neither including the quotient as main effect [*χ*^2^(1) = 0.98, *p* < 0.323] nor as interaction with interactive vs. non-interactive states [*χ*^2^(1) = 0.27, *p* < 0.607] significantly improved model fits for mean interactivity ratings. Similarly, for mean RTs, neither including the quotient as main effect [*χ*^2^(1) = 0.45, *p* < 0.50] nor as interaction with interactive vs. non-interactive states [*χ*^2^(1) = 0.01, *p* < 0.908] significantly improved model fits.

None of the answers to the written open text questions indicated any suspicions about the cover story or any awareness of deceit. In the interview, two participants indicated that during the experiment they developed the suspicion or had asked themselves whether they actually had interacted with the partner they previously had met (both participants were excluded from further analysis, see above).

## Discussion

This study focuses on the question whether and how humans are able to recognize interactivity in triadic interactions. To this extent, we gave our participants two tasks, one in which participants had to observe and recognize gaze states (ObR) and one in which they had to engage in different gaze states (AcR). While the former condition was the actual target condition and basis for the analysis, the latter was necessary to maintain the semblance of a balanced study design suggested by the cover story. As our main result, we can show for the first time that human participants are perfectly able to use gaze cues to judge interactivity by spotting the contingencies between their own and the agents’ behavior without any explicit instructions how to do that. In the analysis of the interactivity ratings, we found that participants consistently and successfully discriminated between interactive and non-interactive states. These findings empirically substantiate the hypothesis of gaze communication being a precursor of human cooperation (Moll and Tomasello, 2007; Tomasello et al., 2007). Findings from phylogenetic and ontogenetic studies support this notion by showing that attending to eyes and communicating via gaze are pivotal steps toward higher levels of social cognition (Tomasello and Carpenter, 2005; Tomasello et al., 2007; Grossmann, 2017). So far, however, these proposals have been hypothetical, i.e., based on phylogenetic and evolutionary considerations. Here, we can explicitly show that gaze is sufficient for humans to establish the experience of mutual interaction as a prerequisite for building social relationships.

We also found differences in the interactivity ratings within interactive-states and within non-interactive states suggesting considerable sensitivity to variations in the tempo-spatial parameters of perceived gaze behavior. Our expectation that a gaze following agent would more easily elicit the experience of interactivity was not confirmed. This hypothesis was based on the assumption that actively following an initiating agent would be more demanding than being followed by a responding agent. Earlier studies had shown that humans innately expect gaze following (Pfeiffer et al., 2011) and perceive the initiation of joint attention as rewarding (Schilbach et al., 2010; Pfeiffer et al., 2014; Oberwelland et al., 2016). However, the present data suggest that agents who initiate joint attention are significantly more readily experienced as interactive than a merely gaze following agent. This might be explained by the fact that responding to joint attention bids might be considerably easier than to actively initiate joint attention. This interpretation is in accordance with phylogenetic and ontogenetic findings suggesting that IJA requires more complex cognition as compared to RJA. For example, chimpanzees are able to follow someone’s gaze but do not initiate joint attention themselves (Tomasello and Carpenter, 2005). Human children acquire the basis of RJA from the early age of 6 month in comparison to the initiation of attention which does not occur before the second year of life (Mundy and Newell, 2007; Mundy et al., 2007).

The non-interactive states OO and PO were significantly more often identified correctly as non-interactive than the INT state. During OO the agent was mainly focused on the objects and looked at the participant only to a lesser extent. Humans are typically very sensitive to how other persons explore and behave in a shared environment. Our perception and processing of objects seem to be fundamentally altered when we observe other person attending to them (Becchio et al., 2008). Objects subsequently appear more familiar (Reid et al., 2004; Reid and Striano, 2005) and likeable (Bayliss and Tipper, 2006; Bayliss et al., 2006). Our results suggest that despite such effects, we are still able to discern that the behavior we observe is not related to us or at least not aimed at us. The same might be true for the PO state. Contrary to our prior hypothesis, participants did not report the PO agent as more interactive than OO, notwithstanding the higher chances of eye contact in these situations due to the agent more frequently looking at the participant. The instructions defined an interaction in terms of mutual and reciprocal responses between both partners. Low interactivity ratings for PO might therefore be just a sign for the participants’ adherence to the instructions instead of disclosing their intuitive, subjective definition of an interaction. Despite that, participants were able to differentiate between an active, reciprocal interaction and person-focused but passive visual attention. This is in line with findings showing that humans are very sensitive to differences in the interactional affordance in the context of more pronounced contrast between encountering real persons as compared to facing static pictures (Hietanen et al., 2008; Pönkänen et al., 2011).

In our experimental setup, INT appears to be the most ambiguous of all states, receiving almost as many interactive as non-interactive ratings. The inward directed attention and thus absence of any obvious attentional focus in the environment probably made it impossible to attribute intentions of interaction. In other words, gaze alone is no longer informative as soon as the interaction partner is in a state of introspection or mind-wandering (see section “Limitations”).

In order to better understand the emergence of the experience of interaction, we analyzed the relationship between the gaze behavior of the participants and the agent’s behavior. We did not find any effect of the agents’ intended interactivity of the encounter on the distribution of the participants visual attention between objects and agent. However, when looking at the synchronization with the agent’s behavior, we found an increase in the number of eye contact instances and joint attention instances in interactive as compared to non-interactive states. Thus, one of the participants’ strategies to judge upon interactivity might have been based on the frequency of eye contact and joint attention instances. The analysis of the effect of the number of eye contact and joint attention instances on the participants’ decisions revealed significant differences between non-interactive and interactive encounters. Importantly, during interactive encounters, the emergence of eye contact and joint attention had much higher effects on the subsequent interactivity ratings. One plausible interpretation could be that participants “tested” the agents’ reciprocity by attempting to establish eye contact and joint attention and subsequently assessing whether the timing of resulting joint contingencies could be attributed to an interacting agent that takes into account the gaze behavior of the participant. Considering the importance of fine-grained timing during such gaze-based interactions it is plausible that the emergence of interactivity is deeply embedded in the temporal enfolding of gaze-based encounters and can only be experienced over time. This is in line with the understanding that non-verbal communication is a dynamic and continuous process (Burgoon et al., 1989) that cannot be fully comprehended through the passive observation of discrete events, uncoupled from the flow of communication.

With respect to the differences in the duration of the decision between the different conditions, we found a correlation between the mean RT of participants and mean correctness scores, suggesting that participants who invested more time were able to make better informed decisions. When comparing RT between states on a single trial level, RTs in non-interactive states showed a pattern roughly corresponding to that of the correctness scores. i.e., RTs reflected the ambiguity and associated difficulty to judge the interactivity. When comparing the participants’ reactions to RJA vs. IJA agents we found longer RTs for the more unequivocal IJA state (as reflected in higher interactivity ratings). One explanation might be that participants needed more time to identify this maximal complex state.

Previous studies about social gaze, even those employing gaze-contingent interactive paradigms, were mostly based on a trial structure that sharply restricted the interaction to a few seconds (Wilms et al., 2010; Pfeiffer et al., 2011, 2012; Oberwelland et al., 2016, 2017). Our findings suggest that such short time intervals are probably not sufficient to establish the full experience of interaction during a spontaneous encounter. Earlier studies circumvented this problem by focusing on “atomic” elements of interaction using an exactly predefined time course of specific behavioral elements and explicitly instructing participants. However, this restriction is not compatible with the implicit and dynamical character of social interactions and thus threatens ecological validity (Risko et al., 2012, 2016; Pfeiffer et al., 2013a; Schilbach et al., 2013).

Overcoming this problem required both theoretically and methodologically new approaches. From a theoretical perspective the SGS provides the holistic framework that is able to encompass and describe the entire span of possible interactive states (Jording et al., 2018). Methodologically this study profits from the development of the new agent-platform TriPy that implements the states of the SGS and allows for a degree of interactional freedom not available with previous setups (Hartz et al., submitted). In combination, these developments allowed us for the first time to investigate the unfolding of a purely gaze based interaction.

### Limitations

Several limitations with respect to the study design need to be considered when interpreting the results. First, we deliberately focused on gaze and restricted all communication to this particular important non-verbal communication channel. The availability of additional channels would certainly have facilitated the establishment of interactions in this study, resulting in more decisive, and faster interactivity ratings. However, the goal of this study was to test explicitly the potential of gaze communication to establish interactions in a way that results can inform studies about non-verbal multi-channel communication. Furthermore, we aimed at studying the individual characteristics of predefined states of gaze interactions and therefore chose a design where the agents displayed only one state at a time. Based on these results it would now be interesting to investigate how transitions between these states might take place (Jording et al., 2018). Therefore, sampling experiences of participants at random time points in an interaction with an agent who dynamically transitions from one state to another might constitute a promising approach.

We did not aim for the systematic investigation of effects of inter-individual differences during the establishment of gaze interactions and while we included a broad age range, we did not balance our sample with regard to gender. In addition, we only used one VC with a male, middleaged appearance and did not systematically match age and gender between participants and agent. Although we did not find any significant effects of age or gender on the quality or timing of the participants’ ratings, we cannot rule out the possibility of any influence. Further investigations controlling for the participants’ age and gender distribution and a systematic matching between participants and agents are required to elucidate this question.

### Conclusion

Results indicate that humans are able to establish gaze interaction without any instructions or additional communication channels, supporting theoretical assumptions of the fundamental role of gaze communication in the development of human social behavior. Our data suggest that human participants are able to identify interactivity not only based on passive observation but potentially by actively studying the agents’ responsiveness based on successfully established mutual eye contact and joint attention. However, participants were not only able to distinguish interactive and non-interactive situations, but behavioral differences between the non-interactive states elicited differential experiences of the interaction. Interestingly, the participants’ performance did not predict their post-experimental assessment of the tasks difficulty. This suggests that decisions were based on intuition or at least partly beyond conscious processing, which corresponds to the presumably implicit and automatic character of non-verbal communication (Choi et al., 2005). An intriguing next step would now be to integrate additional non-verbal communication channels, potentially in a more immersive environment (e.g., a virtual reality), or to investigate the establishment of interactions in cases of impaired communication abilities as in autism spectrum conditions.

## Data Availability

The datasets for this manuscript are not publicly available because the supervising ethics committee has not yet approved the publication of the raw data. Requests to access the datasets should be directed to the corresponding author.

## Ethics Statement

This study was carried out in accordance with the recommendations of the Principles of Good Scientific Practice, ethics committee of the Medical Faculty of the University of Cologne, Germany, with written informed consent from all subjects. All subjects gave written informed consent in accordance with the Declaration of Helsinki. The protocol was approved by the ethics committee of the Medical Faculty of the University of Cologne, Germany.

## Author Contributions

All authors substantially contributed to the conception of the work. AH, MJ, KV, and MS-R designed the study protocol. AH implemented the paradigm code. MJ conducted the pilot study and the main experiment. MJ and AH analyzed the data. MJ drafted the manuscript. AH, GB, KV, and MS-R critically revised the manuscript.

## Conflict of Interest Statement

The authors declare that the research was conducted in the absence of any commercial or financial relationships that could be construed as a potential conflict of interest.

## Footnotes

1 https://www.python.org
